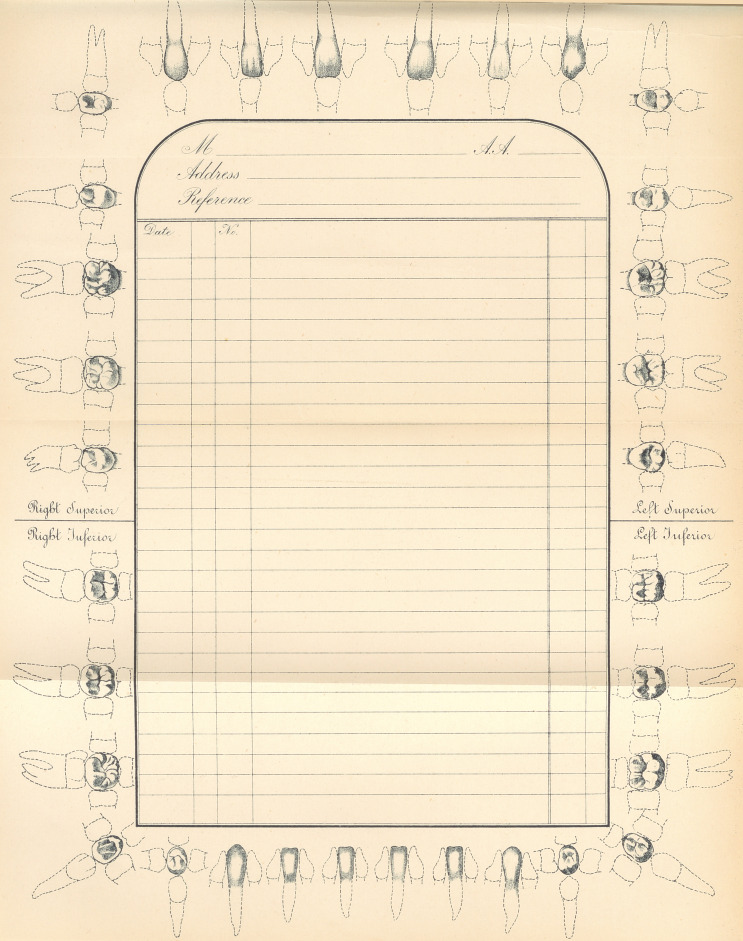# Improved Dental Ledger and Book-Keeping

**Published:** 1890-10

**Authors:** L. C. Bryan

**Affiliations:** Basel, Switzerland


### IMPROVED DENTAL LEDGER AND BOOK-KEEPING.

BY DR. L. C. BRYAN, BASEL, SWITZERLAND.

Copies of the dental ledger have already been passed round. I have a system of keeping books, and I find that every other man has also, and he wants to adhere to his own system, but if there is anything in this sheet which interests you, or if you find any good points, they can be readily copied by any lithographer. There is certainly one advantage, and that consists in having the four sides of the teeth representing the size of the filling if you leave an old one in, and have afterwards trouble with the original filling; it can be successfully designated on your diagram, and you can show your patients how the matter stands on the four sides. In these lithographed stones, which I have had made, each one has had defects more numerous than the first, and I have spent several hours in drawing and improving; still the result is not satisfactory. If I fill a central incisor on the surface, I mark it

*M* ______________________________ *A.A.* __________

*Address* ______________________________

*Reference* ______________________________

| *Date* | | *No.* | | | |
|---|---|---|---|---|---|

Right Superior

Right Inferior

Left Superior

Left Inferior

No. 1, note the operation performed, the material used, and whatever notes I may wish to make. Any number or half a dozen fillings can be recorded in one line. I have an entire method, but I need not call your attention particularly to it. I have the spaces for recording all operations, and figuring up the account. I do not require any credit column where I have a separate cash account. There is no necessity for an elaborate profit and loss account of book-keeping, because my patients in Basel all pay up well.

One soon develops his own system of abbreviation and signs when he wishes to economize time and space. The operations year after year are numbered in order, so that, by following down the columns marked next to the date, we find the various numbers corresponding to those opposite to any tooth or teeth of which we wish to investigate the history. The advantages of a clear chart with self-defined and ample space for pencilling marginal notes in regard to the condition of the tooth, and presenting as soon as the book is opened to the ledger page desired, is an inestimable boon to the busy operator. I have simply Chandler's appointment book and this ledger. At night I take my list of patients from my appointment book and write up each account, the whole notation requiring fifteen to thirty minutes. In that time I have drawn a line over the surfaces filled on each tooth, put a number there, and the same number in the number column, stating material used and the price. By any system of abbreviation two or three operations can be recorded on each line, and valuable notes as to methods and materials be made, besides the marginal notes. In this way fifty operations can be noted on one page,—enough for several years. I have loose pages to paste in when one is full, or when other members of the family come, and I wish to keep them together. One can put two or three accounts on one page when there is a good class of teeth, which need new operations.

When I fill a root I show on diagram just how far down each root is filled, so that, in case of after-trouble, I know where it has originated.

If the roots are recorded as properly treated and filled, I know my treatment must be external. If I repair an old filling, the repair can be clearly shown with ample diagram spaces, and in case of failure the patient can perceive just how far I am responsible. With a record of all operations performed the interest is stimulated in observing and comparing methods and materials, and the pleasure in practice is enhanced. The diagram simplifies any system, and keeps all operations clearly exposed for reference. These notes I

often make while waiting for the servant to arrange the operating-room between patients. I note the date of the sending of the bill, and when paid, on one line below the account, and so have all my records here, not even needing a cash-book. In my appointment book, opposite the name, I note the amount of the daily operation for each patient, in characters, as a merchant marks his goods with a cost mark; and so can readily figure up my income when desired.

I have been several years arranging this diagram, and in October, 1888, wrote to a friend in Boston, telling him of my plan, and asking if any such system existed in America. He did not answer until May, 1889, when he enclosed a sheet of a similar ledger page, which he had in the mean time arranged and had copyrighted this year (1889).

## Figures and Tables

**Figure f1:**